# A Focused Ethnographic Study of Sri Lankan Government Field Veterinarians’ Decision Making about Diagnostic Laboratory Submissions and Perceptions of Surveillance

**DOI:** 10.1371/journal.pone.0048035

**Published:** 2012-10-25

**Authors:** Kate Sawford, Ardene Robinson Vollman, Craig Stephen

**Affiliations:** 1 Farm Animal & Veterinary Public Health, Faculty of Veterinary Science, University of Sydney, Camden, New South Wales, Australia; 2 Department of Ecosystem and Public Health, Faculty of Veterinary Medicine, University of Calgary, Calgary, Alberta, Canada; 3 Department of Medical Sciences, Faculty of Medicine, University of Calgary, Calgary, Alberta, Canada; 4 Department of Community Health Sciences, Faculty of Medicine, University of Calgary, Calgary, Alberta, Canada; Federal University of Pelotas, Brazil

## Abstract

The global public health community is facing the challenge of emerging infectious diseases. Historically, the majority of these diseases have arisen from animal populations at lower latitudes where many nations experience marked resource constraints. In order to minimize the impact of future events, surveillance of animal populations will need to enable prompt event detection and response. Many surveillance systems targeting animals rely on veterinarians to submit cases to a diagnostic laboratory or input clinical case data. Therefore understanding veterinarians’ decision-making process that guides laboratory case submission and their perceptions of infectious disease surveillance is foundational to interpreting disease patterns reported by laboratories and engaging veterinarians in surveillance initiatives. A focused ethnographic study was conducted with twelve field veterinary surgeons that participated in a mobile phone-based surveillance pilot project in Sri Lanka. Each participant agreed to an individual in-depth interview that was recorded and later transcribed to enable thematic analysis of the interview content. Results found that field veterinarians in Sri Lanka infrequently submit cases to laboratories – so infrequently that common case selection principles could not be described. Field veterinarians in Sri Lanka have a diagnostic process that operates independently of laboratories. Participants indicated a willingness to take part in surveillance initiatives, though they highlighted a need for incentives that satisfy a range of motivations that vary among field veterinarians. This study has implications for the future of animal health surveillance, including interpretation of disease patterns reported, system design and implementation, and engagement of data providers.

## Introduction

New diseases in animals and people are being identified more frequently than ever before and this trend is expected to continue [1]. It has been estimated that between 60 and 75 percent of emerging infectious diseases (EID) in people have arisen from animals [2]–[4]. Recent investigations have implicated increasing demand for animal protein, expansion of intensive animal agricultural systems, long-distance transportation of live animals, consumption of wild animals, and habitat destruction as important drivers behind EID events [2], [4]. Risk maps based on socio-economic, environmental and ecological variables that correlate with past EID events suggest that areas at highest risk for future EID events are most concentrated in lower-latitude, low-resource countries [5]. Preventing and containing the impacts of EIDs necessitates early EID detection and response in animal populations [6]. As part of the response to this need the practice of animal surveillance is changing rapidly [7].

Historically, animal infectious disease surveillance systems have revolved around diagnostic laboratory sample submissions, including those collected as part of active and passive surveillance [8]. Surveillance of submissions to laboratories will continue to be an important component of any surveillance system because, for many infectious diseases, laboratory diagnostics are the only way to make an etiologic diagnosis that can inform control and policy responses. However, in the case of EIDs, diagnostics may not exist for novel or previously unknown pathogens, making surveillance systems reliant on other data. Moreover, not all potential cases of infectious disease are submitted to laboratories. In the domestic animal health field there is a series of selection biases that affect which cases are submitted for diagnostics. Veterinarians play a critical role in determining which cases will be submitted for diagnostics and their process of case selection, in combination with direction from animal owners, influences the types and amounts of samples assessed at the laboratory. The result is the potential introduction of sampling bias that will affect disease patterns described by laboratory-based surveillance [9]. In order to understand the impact of bias on laboratory-based surveillance data, submission patterns of veterinarians and the factors that influence their decision to submit samples must be better understood [9]–[11].

Surveillance systems that include pre-diagnostic data generally have the aim of identifying disease outbreaks earlier than would have been possible with laboratory-based surveillance data alone [12]–[18]. Focus is diverted away from etiological or definitive diagnoses and onto patterns in clinical signs or syndromes. The initial step in realizing the potential of these methods in high-resource settings is securing access to appropriate data [12], [19]. In low-resource settings, where digital storage of information is limited, the initial step is often to engage various subsections of the health care community to provide necessary data [20]. Within the animal health field it may be veterinarians or para-veterinarians in partnership with farmers that provide healthcare services to domestic animals. In lower-resource settings where veterinarians provide these services they have access to animals and are frontline observers of signs of disease, making them an ideal source of pre-diagnostic data. A number of animal disease surveillance systems rely on veterinarians to provide clinical case data [21]–[26]. Therefore, understanding the attitudes and perceptions of veterinarians as they apply to surveillance is foundational to the development and assessment of surveillance systems in low-resource settings that utilize clinical case observations from veterinarians as a data source.

Qualitative research methods are being increasingly used in low-resource settings to identify factors that impact the uptake and application of health-related ideas, technologies, and practices [27]–[31]. They have also been employed in the human health field to explore the use of health data in public health practice, as well as factors that act to facilitate or hinder use of these data [32]–[34]. However, qualitative research papers that explore the utilization of laboratory services by veterinarians or para-veterinarians in low-resource countries were unavailable at the time of this research. This paucity of information precludes confident assessment of the representativeness of animal infectious disease occurrence data in many low-resource countries where reporting is based on laboratory submissions. In addition, there were no qualitative research papers available that investigate the ability and willingness of veterinarians or para-veterinarians in low-resource countries to participate in clinical case reporting as part of a surveillance system. This represents a significant deficit in the literature given the call for increased reporting of clinical case data for the purpose of infectious disease surveillance in low-resource countries [35].

In this paper we report the results from a focused ethnographic study that aimed to advance understanding of the factors that influence government field veterinarians in Sri Lanka to submit cases to a laboratory, and to describe their perceptions of infectious disease surveillance, including the complex of factors that impact their ability and willingness to participate in surveillance initiatives that depend on clinical case submissions. The results reported here can inform interpretation of infectious disease patterns reported by veterinary laboratory-based infectious disease surveillance systems in low-resource settings and improve efforts to engage veterinarians in future surveillance initiatives.

## Methods

### Ethics Statement

The Conjoint Faculties Research Ethics Board at the University of Calgary approved the study proposal (file number 4530).

### Study Design

In focused ethnography, research is not directed towards a culture but rather a particular subculture or group of participants that share some feature or features [36]. The term “focused ethnography” describes a research approach employed when what is sought is an explication of behaviour or beliefs pertaining to a specific area so that their meaning among a defined group of individuals might be understood [36]. This method is employed when research questions are best responded to through descriptive analysis and interpretation [36].

This study consisted of in-depth interviews with participants linked by their experience as field veterinary surgeons (FVS) employed by the Department of Animal Production and Health (DAPH), a national-level body responsible for control of livestock diseases, livestock research, animal breeding, and education in animal husbandry in Sri Lanka. A brief description of the laboratory capacity available to FVSs in Sri Lanka provides contextual information necessary for interpreting data collected during the interview process. In-depth interviews were conducted in January 2010.

### Study Participants

Eligible FVSs were those who participated in the Infectious Disease Surveillance and Analysis System (IDSAS). The IDSAS is a mobile phone-based surveillance system that was piloted in Sri Lanka in partnership with the DAPH during 2009. It enabled FVSs to submit data concerning animal health-related events from the field on a daily basis [25]. Refer to [25] for details on how IDSAS participants were selected. All forty FVSs who took part in the IDSAS consented to the interview process. All FVSs spoke English as all attended the Faculty of Veterinary Medicine and Animal Science at the University of Peradeniya where the curriculum is delivered in English. However, there was variation in English proficiency between potential participants. When the IDSAS pilot project began participating FVSs were characterized by age, year of graduation from veterinary school, years as a FVS, sex, divisional secretariat (DS) division, and district practice location. Twelve of the forty FVSs were later purposively selected to participate in the interview process with the aim to construct a group of participants with maximum demographic variation in the characteristics listed previously. To facilitate the interview process a high degree of proficiency in spoken English and English language comprehension was required as an attribute common to all participants. Descriptive statistics were used to summarize the characteristics of the study participants.

### In-depth Interview Structure

Participants were asked at the beginning of the interview to confirm orally that they had signed the informed consent and agreed to be audio-recorded. Each in-depth interview, conducted in person by KES, was no longer than 2 hours in length. A semi-structured format with a series of standardized open-ended questions was used (Table 1). An initial set of follow-up probes was drafted and employed where appropriate: the purpose of the probes was to delve into participants’ individual responses and therefore probe inclusion and exclusion, specific wording, and order in which they were asked varied between interviews. The lead-in question to each topic remained the same for each interview however probes evolved as subsequent interviews were conducted (Table 1).

**Table 1:** Open-ended questions and follow-up probes used during in-depth interviews.

Topics
Lead-in question and follow-up probes
Decision making around laboratory submissions
Please describe the various factors that affect your decision to submit samples for diagnostics
What do you see as the benefits of laboratory confirmation?
What are the costs of sample submission?
Are there instances where laboratory testing is more warranted – or less warranted? What influences this?
When it comes to sample submission who is the final decision maker in the process?
What kind of value does laboratory testing provide?
Are there types of cases in which you feel laboratory testing is more urgent?
Do you have particular ‘flags’, ‘indicators’, or scenarios that prompt you to consider laboratory testing more carefully?
How does your familiarity with the species or syndrome affect your decision?
Do you think your decision-making process behind the submission of samples to labs has changed over time?
Do you think you’re submitting the same types and numbers of cases to laboratories as you were when you started in practice?
How many diagnostic tests are you running in your clinic versus submitting to an outside lab?
Participation in disease monitoring and surveillance
Please talk to me about how willing you think veterinarians are or would be to participate in a disease monitoring and surveillance program
What are the obstacles to participation?
What are the potential benefits?
Is there conflict between the different roles veterinarians are supposed to play and the interests they are compelled to adhere to or represent?
Should veterinarians be more engaged in disease monitoring and surveillance? If yes, how might this be accomplished?
Do you think veterinarians have additional infectious disease information to provide that may be missed by diagnostic laboratory based surveillance?
Disease monitoring and surveillance and interactions with farmers
Do you discuss disease monitoring and surveillance with farmers?
Please talk to me about the range of attitudes you encounter, using specific examples wherever possible
How do you address concerns farmers have about the consequences of infectious disease identification?
What do you see as the potential benefits to such conversations?
What do farmers see as their role in disease monitoring and surveillance or do they see themselves as having a role at all?
How concerned about the potential for disease outbreaks do they appear?
How do you think farmers could be better engaged in disease monitoring and surveillance?
Are there other members of the community that could be more effectively engaged in disease surveillance?

All in-depth interviews were recorded using two digital audio recorders. At the end of each interview the recordings were downloaded onto a password-protected laptop computer. Both audio files were reviewed to ensure the interview had been recorded in its entirety. There was only one month available to conduct all of the interviews and therefore analysis of the audio files was not possible between interviews. Audio files were reviewed between each set of interviews in the four districts to inform probes used in subsequent interviews and to allow interviews early in the research process to inform those that came later. Interview data collection ceased after completion of the twelfth interview. Interviews were transcribed verbatim from the audio recordings by a professional transcriptionist at the end of the interview data collection period. Personal identifiers were removed from the transcribed files to ensure participants’ responses remained anonymous. A single copy of each original audio file was transferred onto two password-protected DVDs, which were stored together in a locked cabinet in a locked office according to University of Calgary policy. All original audio files were then removed from the laptop computer. Data were analyzed after transcription of the interview audio files.

In qualitative research data saturation is defined as the completion point of the data set and results when there is data replication or redundancy, when there are no new information or themes emerging from subsequent interviews, and when the categories, themes and relationships among them are thoroughly described [37]. In studies that ask questions similar to the ones posed in this study, six in-depth interviews usually allows for data saturation, while when twelve in-depth interviews are performed data saturation is almost always attained [38].

Collection of interview data concluded after the twelfth interview due to time constraints. KES was in Sri Lanka for a limited time period and it was not possible to conduct further interviews or analyse the data prior to departure. Other reasons for terminating data collection include: data saturation has been achieved; a lack of available individuals who met the study inclusion criteria; and budgetary constraints [39]. The travel time between districts, the limited time available for interview data collection, and the desire to achieve data saturation were the drivers behind the sample size of twelve.

There were a number of data sources accumulated in addition to the in-depth interview transcripts: memos were made by KES to document decisions made in the data collection and analysis process, day-to-day activities, and any comments concerning methodology; a reflective journal was kept by KES, further describing the research process and the researcher’s experience with participants; and fieldnotes were used to record any observational data. Memos and the reflective journal were captured directly in Microsoft Word while fieldnotes were hand-written onto the interview guide during each interview and later transcribed. All raw data and material arising from the research activity were scanned into electronic files and the original documents destroyed. Dr. Craig Stephen, Principal Investigator and Doctoral Supervisor, is storing the electronic version of these materials for seven years as required by the University of Calgary’s Faculty of Medicine Research Policy Guidelines for Integrity in Scholarly Activity.

### Data Analysis

The first step in data analysis involved reading through the interview transcripts and coding the data by interview question using QSR International’s NVivo 9 (N9), a qualitative analysis software suite that enables researchers to organize and retrieve qualitative data, including textual material. Thematic analysis [40] was then performed on the transcripts. During this process data were systematically organized within N9 using codes that KES inductively derived from the records. The goal was to identify concepts, categories, relationships, and themes. Concepts are the basic units of analysis. During identification of concepts, the central meaning of each piece of transcribed text was described in a short statement, referred to as a code [40]. Concepts were grouped in categories, groups of content that share common features. Similarly, categories were organized around themes. Creating themes is a way of linking underlying meanings that reoccur within categories [40]. Individual categories and themes were described by a code [40]. All of the codes were reviewed to ensure the concepts, categories, themes, and relationships between them were completely and appropriately described. All data presented in the results section reflect the observations, insights, and opinions expressed by participants.

During the interviews the meaning of the conversation was more evident than indicated by the transcripts because of accompanying facial expressions and gestures [41]. In order to convey the information in clear language and allow accounts to flow smoothly, the participants’ wording within quotes included in this paper has been edited carefully to make quotes easier to understand while preserving their meaning. This approach is respectful because participants were educated people that would be more articulate if they were speaking in their first language; this approach is deemed necessary and appropriate in qualitative research when participants speak English as a second or third language [42], [43].

## Results

### Veterinary Diagnostic Laboratory Infrastructure in Sri Lanka

The DAPH carries out surveillance for World Organisation for Animal Health-listed diseases and EIDs in animals. The Veterinary Research Institute (VRI) operates under the DAPH and is the only national-level government organization in Sri Lanka that provides veterinary laboratory services. District-level laboratory diagnostics are provided by Veterinary Investigation Centres (VIC) located in the following districts: Anuradhapura, Badulla, Hambanthota, Chillaw, Jaffna, Matara, Peradeniya, Rannala, Polonnaruwa, Ratnapura, Vaunia, Welisara, Kegalla, Nuwara Eliya, and Dambulla. This represents a subset of the twenty-five districts in Sri Lanka. Each VIC is headed by a Veterinary Investigation Officer (VIO), a senior veterinarian with experience working as a FVS. One aim of the DAPH is to establish VICs in every district in Sri Lanka. All participants worked in districts in which there was a VIC. For a description of the government veterinary laboratory capacity in Sri Lanka refer to [44].

### Study Participants

Each of the twelve participating FVSs practiced veterinary medicine in a distinct DS division. Three came from the Matara district; three from the Anuradhapura district; three from the Nuwara Eliya district; and three from the Ratnapura district. For a map of the study districts refer to [25]. Participants ranged in age from 33 to 54 years (median, 37 years; mean, 39.5 years); 7 were male (58%). Participants graduated from veterinary school between 1984 and 2003 (median, 1999; mean, 1997). Participants had from 2 to 24 years (median, 5.5 years; mean, 7.96 years) of experience as a FVS within the DAPH. Further details about the study participants are not provided to protect their identities.

### Overview of the Themes and Categories

Themes and categories are summarized in Table 2 and linked to the research aims of this study.

**Table 2:** Research aims linked to the themes and categories that emerged during data analysis.

Research aims
Themes
Categories
Advance understanding of the factors that influence field veterinary surgeons in Sri Lanka to submit cases to a laboratory
Field veterinary surgeons’ interactions with laboratories
The reported frequency of submissions
Cases from which samples were submitted
The tools employed in making a diagnosis
Perceived benefits of laboratory assistance
Desire for further laboratory capacity
Future laboratory submissions
Factors underlying the frequency of case submissions to diagnostic laboratories
Farmer-level factors
Field veterinary surgeon-level factors
Factors related to veterinary services and infrastructure
Describe field veterinary surgeons’ perceptions of infectious disease surveillance
Field veterinary surgeons and surveillance
Perceptions of the role and value of surveillance
Perceived limitations of current surveillance methods
Willingness to participate in surveillance initiatives
Challenges to surveillance methods that rely upon field veterinary surgeons to submit pre-diagnostic data

### Theme One: Field Veterinary Surgeons’ Interactions with Laboratories

When asked the question ‘describe the various factors that affect your decision to submit samples for laboratory diagnostics’, participants did not supply the information requested. In trying to answer this question, participants focused not on decision-making related to sample submission but rather on how they interacted with the laboratory system in Sri Lanka. There were six categories identified that relate to FVSs’ interactions with laboratories: the reported frequency of submissions; cases from which samples were submitted; the tools employed in making a diagnosis; perceived benefits of laboratory assistance; desire for further laboratory capacity; and future laboratory submissions (Table 2).

#### The reported frequency of submissions

When participants were probed specifically about the frequency of laboratory submissions over the previous year, responses typically ranged from one sample per year to one sample per month, though some said they could not specify a number of samples. A few participants referred to sending samples to the VRI in general terms but only one cited a case where samples were sent to the VRI for the purpose of vaccine preparation. Some participants stated that they had never sent samples to the VRI. When participants talked about specific cases, they often referenced VICs as the laboratory to which they submitted samples.

#### Cases from which samples were submitted

Participants discussed types of cases from which they submitted samples: they sent samples when there was disease spread or when diseases were highly contagious; when the initially prescribed treatment proved ineffective; to gain knowledge; and when a notifiable disease was suspected. Participants reported that they employed means other than laboratories when they made diagnoses.

#### The tools employed in making a diagnosis

The history, clinical signs, and physical exam findings guided participants’ approach to cases and clinical diagnoses. Obtaining a history was described as frustrating since a large percentage of farmers failed to keep animal health and production records.

Some participants provided an example of a list of differential diagnoses for particular case presentations. Participants talked in general terms about basing their diagnoses and treatment choices on the observed clinical signs, and gave specific case examples. They emphasized that previous experience informed this process. One participant explained that for most cases in their clinic they made a diagnosis and supplied medications to farmers based on the reported history and clinical signs, rendering travel to the farm unnecessary.

Some participants had no laboratory tests available in their clinic, while others were able to run the California Mastitis Test (CMT). Microscopes were available in some clinics for blood smear examination, however only one was equipped to stain blood smears. A couple of participants have utilized laboratories outside of Sri Lanka’s government veterinary laboratory services, including a nearby human laboratory and college.

Participants consistently referenced post-mortem examination (PME) as part of their diagnostic process, particularly for poultry A few participants indicated they had performed PMEs on cattle.

Several participants stressed the importance of the response of the animal to therapy: it was discussed as part of making a clinical diagnosis and guided their approach to future cases.

I think that if my diagnosis is correct and my treatment is correct the animal will recover. There may be cases where my diagnosis is wrong but the treatment is correct and that is what is important. (Interview 2, Lines 381–387)

Some participants would follow up with the farmer one or two days after administering treatment to find out if the animal’s condition had improved: in others cases if an animal did not respond to an antibiotic participants switched to a different antibiotic.

As part of their diagnostic process, participants referred cases to the VIC or VIC, called the VIO for advice, or had the VIO come to assist with a challenging farm or case. Other reported options were to consult with faculty veterinarians at the University of Peradeniya, share successful case outcomes with colleagues at meetings, and use means outside the animal health system (e.g., public health inspectors, police) for urgent situations (e.g., an animal head needed to be submitted for rabies testing), and taking part in emergency response teams that were able to quickly contact others in the case of an infectious disease event.

#### Perceived benefits of laboratory assistance

Participants indicated that diagnostics are valuable in that they can identify the agent, confirm the clinical diagnosis, and inform treatment. Some participants talked about the types of laboratory support that were helpful in particular cases: antibiotic sensitivity testing in mastitis cases; yogurt and curd cultures in suspect *E. coli* cases; vaccine preparation in wart cases; bacterial culture in suspect salmonella outbreaks; and diagnostics in suspect viral or bacterial etiology cases.

#### Desire for further laboratory capacity

All participants indicated they would like the ability to perform further diagnostics: some talked about the types of diagnostics they would like to run; others talked more about the types of diseases for which they would like to test; and a couple talked only in general terms about diagnostics. Named diagnostics and targeted case presentations included: mastitis; bacterial culture and antibiotic sensitivity testing; brucellosis testing; cases for which there is no clinical diagnosis; testing for parasitic disease; blood calcium measurement; leukocyte counts; blood testing generally; cases in poultry; infectious disease cases; unusual cases; and cases where there is sudden morbidity and mortality, particularly in poultry. One participant expressed a desire for additional in-clinic diagnostics including: the ability to measure [parasite] eggs per gram of feces; create, stain, and examine microscope slides; quantify leukocytes; and measure packed cell volume in the clinic.

#### Future laboratory submissions

Participants discussed future circumstances under which they intended to submit samples, including suspected notifiable disease cases (e.g., black quarter (*Clostridium chauvoei*), foot and mouth disease (FMD), and brucellosis (*Brucella abortus*)), cases of high mortality in poultry, and cases where chicken and cattle had the same clinical signs. As an alternative to sending samples, some participants would send farmers directly to the laboratory.

### Theme Two: Factors Underlying the Frequency of Case Submissions to Laboratories

Factors that provide explanation for the frequency of case submissions to laboratory occurred at one of three levels: the farmer; the FVS; and veterinary services and infrastructure (Table 2).

#### Farmer-level factors

Factors that help account for the limited number of case submissions to laboratories that operate at the level of the farmer are: notification of a FVS of an animal health-related event; delivery of samples to a laboratory; level of education; and farmers’ sources of income and economic status.

Participants reported that farmers generally contacted a FVS when they had an animal-related concern, and when an animal was sick. However, farmers were not always concerned about an animal death. For example, in the case of deaths in a poultry flock, farmers would bring birds for PME to the clinic; however, more experienced poultry farmers would sometimes decline to bring dead birds to the clinic because pharmacies would provide pharmaceuticals to the farmer without a prescription. In cases of deaths in large animals, farmers often buried carcasses without informing a participant. When deaths were brought to the attention of a participant, the cause of death could remain unknown.

We have had one or two cases of generalized edema in cattle. The animal gets bigger and bigger by the day and within two to three days they die. Everything, the head as well as the legs, is edematous. Still we are not sure what they are dying of. (Interview 4, Lines 246–253)

Some farmers were reluctant to deliver samples to the laboratories, even after samples were collected.

In the last month I’ve recorded two or three cases of rabies in large animals. However, the farmers did not bring the heads to {town name}. It is a large head, no? They don’t like to cut and bring the head but based on the clinical signs we decided it was rabies. (Interview 12, Lines 333–340)

Dairy farmers in particular had limited ability to leave the farm. Laboratory staff, veterinary office staff, or participants themselves may have transported samples if the farmer was unable to do so.

The education level of farmers presented a challenge to participants because of language barriers (i.e., different dialects) and illiteracy.

Some farmers are coming and saying that the cow is not chewing the beetles but we know from experience that they mean the cow is not eating and not regurgitating […]. (Interview 2, Lines 617–624)

Participants and farmers sometimes had different ideas about clinical presentations that indicated a serious animal health condition.

Often domestic animals supplemented a farmer’s primary source of income (e.g., tea cultivation) and farmers were very poor, both of which impacted farmers’ ability to deliver samples.

#### Field veterinary surgeon-level factors

Factors that explain the limited number of laboratory submissions that operate at the FVS level include: treating based on an animal’s clinical signs; making a clinical diagnosis and administering treatment based on that diagnosis; confidence in clinical diagnoses; knowledge of laboratory capacity in Sri Lanka; and failure to conduct a PME.

Participants treated signs of disease in the absence of a clinical diagnosis:

It is mainly in poultry cases that I cannot identify the cause of disease. If the farmer doesn’t like to go to the diagnostic laboratory I treat with broad-spectrum antibiotics. I probably have 20 cases in poultry a year in which I haven’t identified a proper cause and I primarily treat the symptoms. Sometimes a farmer will only bring one carcass and it will be normal but the other birds still require treatment. In those cases we blindly administer treatment. (Interview 10, Lines 421–434)

Participants talked frequently about making a diagnosis based on an animal’s clinical signs, and treating based on that diagnosis.

Participants were confident in their clinical diagnoses even though they were not always correct. The potential for misdiagnoses when applying their diagnosis process was discussed:

I remember one rabies case in a cow when on the first day I didn’t think the clinical picture fit with rabies. […] The farmer told me the cow had eaten the jackfruit. I told the farmer that because the cow had eaten the jackfruit, it was straining and had bloat. The farmer didn’t notice there was a dog bite. […] I treated for an indigestion problem. […] On the third day the animal collapsed and was straining much more strongly. Then I saw tearing and salivation, all the rabid signs were present. Then […] the farmer told me there was a dog bite and rabies was confirmed. (Interview 3, Lines 270–281)

Collectively participants referred to the capacity of VICs to undertake bacterial culture; antibiotic sensitivity testing; the CMT; blood and fecal parasite identification; the Rose Bengal Plate Test (RBPT); highly pathogenic avian influenza (HPAI) virus testing; and vaccine production. A few participants talked about the VRI and its diagnostic capabilities, referring to leptospirosis testing, bacterial culture, polymerase chain reaction, and enzyme-linked immunosorbent assay in particular. In contrast, other participants indicated they lacked knowledge concerning diagnostics available at the VRI and have no interactions with the laboratory.

There were a number of different scenarios described by participants during which they did not perform a PME. In the case of death in cattle, a number of participants referred to circumstances under which a PME was not possible and highlighted a lack of necessary equipment as an impediment to the procedure. Distance was also a factor: FVSs have to travel to cattle farms to conduct a PME. In some instances, the carcass was too stiff for PME. Sometimes farmers did not want participants to open up carcasses: at least one participant would insist on PME when animals were insured.

#### Factors related to veterinary services and infrastructure

There are factors that help account for the limited number of case submissions to laboratories that are related to veterinary services and infrastructure in Sri Lanka: costs of animal health care; limited availability of supplies, equipment and facilities; and logistical issues related to sample submission.

Several participants talked about the costs of diagnostics and veterinary services in Sri Lanka. There were marked differences in the cost to farmers reported by participants: there were several payment schemes that depended on the time of day, the availability of government vehicles, and the particulars of the situation. Participants worked normal business hours, but after those hours were able to run private practices. Farmers might have had to pay for diagnostics but this depended on the type of test and the laboratory administering the test. They paid for travel costs if a government vehicle was unavailable. In some instances farmers were charged for drugs only. However, one participant reported that farmers paid for everything: transportation, drugs, and professional fees.

Several participants discussed the limited availability of supplies, equipment, and facilities. They referred to a range of items: blood containers; chemicals for diagnostics; pharmaceuticals (including but not limited to antibiotics); surgical instruments; computers; internet access; potable water; facilities for washing; telephones; and fuel.

Several participants referred to a range of logistical issues related to sample submission, including limited veterinary office staff to transport samples and the tendency for damage to samples during transport. Farmers often made use of bus service to travel, which presented a challenge.

If I visit a case at two or three in the afternoon I must either bring the sample back to the clinic to refrigerate or send the sample directly with the farmer. However […] the farmer can go but he can’t come back because there is no transportation [as bus service does not operate in the evening]. (Interview 2, Lines 22–28)

Participants talked about transportation issues at multiple points during the interviews. Some talked about the issue generally, saying things like “it is difficult for farmers to travel” or “transportation is poor”, while others were more specific, reporting they lacked a government vehicle, had access to a vehicle for a limited number of days in a month, or could travel only a given distance in a month. Those participants without a vehicle indicated that farmers needed to supply some form of private transportation to permit travel to and from farms. Reported travel times from farms to the VIC within a DS division ranged from an hour to four hours in one direction while travel times from farms to the VRI ranging from three to eight hours. A couple of participants stated specifically that they do not know how to address transportation-related challenges when samples need to be sent for diagnostics.

### Theme Three: Field Veterinary Surgeons and Surveillance

FVSs and surveillance occurred as a theme in the data, around which were four categories: perceptions of the role of surveillance; perceived limitations of current surveillance methods; willingness to participate in surveillance initiatives; and challenges to surveillance methods that rely upon FVSs to submit pre-diagnostic data (Table 2).

#### Perceptions of the role and value of surveillance

All participants discussed the role and value of disease surveillance: they talked about it being important to situational awareness, in particular to understand disease conditions encountered by FVSs. Some talked about how surveillance could inform their knowledge and veterinary service activities, including farmer education. Many participants made reference to understanding geographical variation in disease occurrence, which according to one participant could inform disease treatment, while a couple referred to temporal variation, which could guide farm education. Surveillance was reported as important for permitting a rapid response to future highly pathogenic disease conditions.

Several perspectives on surveillance were presented including: it is a duty and the main job of veterinarians; it can provide scientific evidence to underpin clinical practice; it is of economic importance in terms of eradicating disease and saving money on vaccination programs; and it can identify zoonotic disease and is therefore important to human health.

Participation in surveillance initiatives was seen as important to building networks, understanding the current animal health situation in Sri Lanka, and having a good communications system in the event of a future highly pathogenic disease condition. Having good relationships with the private sector, industry organizations, and other government departments and ministries was also viewed as necessary. Networking farmers together was perceived as important to disseminating information about disease.

Participants expressed the belief that the best way to engage farmers in surveillance was through education. Prevention and treatment of more common diseases, contagious diseases and how to protect animals, signs of disease for which to monitor, the need to report clinical signs, and animal management were suggested as topics relevant to farmers. In the case of HPAI, mode of disease transmission, the time it takes for clinical signs to develop and for animals to die, how to protect people from contracting the disease, and the potential consequences of human infection, were highlighted as important knowledge areas for farmers.

A number of participants talked about engaging members of the public in disease surveillance by conducting HPAI training programs in schools and teaching members of the public about risky diseases including leptospirosis, rabies, and HPAI. In contrast, one participant indicated it was important to be cautious about the information you provide to the public about infectious disease because individuals could panic and not eat animal products, which would destroy industries.

#### Perceived limitations of current surveillance methods

Participants believed there were limitations to currently employed disease surveillance techniques. When asked about surveillance programs based on laboratory submissions compared to those based on inputs from FVSs, several participants talked about the fact that many cases are not submitted to laboratories.

We are sending a very small number of samples. I think comparing VIC data with cases seen by veterinarians the big difference is there. (Interview 10, Lines 649–651).

Another participant observed clinical case data sent by FVSs would contain errors, but was stilly worthy of submission.

I can’t submit samples most of the time so the labs are not getting any samples so there would be zero value in the lab data. […] Sometimes there may be some misinterpretations, some more misdiagnoses by the vet, because *E. coli* infection and salmonellosis, they would be two different things with the same solution the veterinarians are submitting. There would be some guesses […] Submissions from veterinarians may be wrong compared to the lab but it has some value […] It is okay to have the FMD cases in his report other than not reporting. It may be something like FMD, but not FMD, but the veterinarian is suggesting that he is suspecting FMD. It is better to have that mistake, other than have him not report the FMD suspected cases. (Interview 2, Lines 505–513)

Participants highlighted drawbacks of surveillance initiatives carried out by VICs, including the sampling of clinically normal cows and the small number of farms included.

#### Willingness to participate in surveillance initiatives

A willingness to participate in surveillance was common to all participants. There was discussion of incentives to encourage participation, with emphasis on monetary compensation. Computer and internet access could serve also as an incentive. Some participants indicated that improvements to infrastructure promote surveillance.

Okay, the financial compensation is good but the thing is you can’t go into the field without a vehicle. Financial compensation will help to encourage the vet but there are problems with infrastructure, at the same time we have to improve the infrastructure.’ (Interview 2, Lines 457–467)

Participants noted that information from surveillance could serve as a form of positive feedback because summaries of cases treated provide a measure of industry impact, though would not be sufficient for all involved in the absence of monetary compensation. Helping farmers reduce their expenses and increase their income was not only reported as a FVS’s duty, but also provided job satisfaction.

#### Challenges to surveillance methods that rely upon field veterinary surgeons to submit pre-diagnostic data

Some participants indicated there were no difficulties in participating in disease surveillance, while others admitted that they sometimes forget to bring instruments for surveillance into the field. Personal factors impact an individual’s participation.

Some persons who were in this program, they wouldn’t have helped you in the data collection. That is a personal thing, that is the nature of the people. […] Maybe the reason for less cases from a particular range is they have shown less interest in data collection, […] Some people they were really interested and they were willing. Some people didn’t have time and some people they can’t correct. (Interview 6, Lines 547–560)

One participant stated that FVSs are a little more interested, and feel a stronger sense of obligation, when the request comes from a foreigner. Some participants felt electronic forms of surveillance were preferable because fewer cases are omitted during the recording process, while one participant admitted that new technology was sometimes difficult to learn. Some participants expressed opinions on the topic of their time and surveillance: the time required for data submission was raised an issue, though it was highlighted that when FVSs have an interest in surveillance they will dedicate time to it.

Following analysis of the twelve interviews the codes, concepts, categories, relationships, and themes were reviewed. The authors observed that there was data redundancy and the categories, themes and relationships between them were thoroughly described. It was also noted that though the last few interviews enriched the data set, they led to no new information or themes. Therefore it was determined that data saturation had been achieved.

## Discussion

Study participants submitted cases to laboratories so infrequently that it is difficult to draw any conclusions about the factors that influence submissions to laboratories by FVSs in Sri Lanka. Participants were able to describe future circumstances under which they would send samples, however there were discrepancies between future intentions and past reported submissions. Participants approached clinical cases in ways that did not include laboratories. This approach, combined with factors at the level of the farmer, and related to infrastructure and delivery of veterinary services in Sri Lanka, helps account for the difference between past case submissions and future intentions to submit samples and contributes to the potential for missed EID events. While participants talked in detail about the role of surveillance and the limitations to techniques currently utilized in Sri Lanka, they indicated a willingness to participate in initiatives that rely on FVSs for data, particularly if they include a variety of incentives.

### Field Veterinary Surgeons’ Interactions with Laboratories

Previous work in Sri Lanka has quantified the small number of submissions to government veterinary laboratories in relation to the caseload of FVSs [44]. This data deficiency, combined with the lack of describable case selection process by FVSs for diagnostics, has implications if laboratories are to be relied upon for EID event detection. Based on the range of factors that contributed to the infrequency of case submission by participants, future changes in clinical caseload are unlikely to be reflected in the number of laboratory case submissions. Deficiencies in infrastructure make simply increasing the volume of diagnostics performed by laboratories unfeasible, in particular at the VRI, which is equipped to perform more advanced diagnostics. In the absence of a substantial number of case submissions from the field, laboratories are highly unlikely to detect a change in disease burden or receive submissions from individual cases of an EID [11].

Though all participants indicated they would like the ability to perform additional diagnostics, there was no consensus on the types of cases that benefit from diagnostics or case characteristics that would drive future laboratory submissions. The discrepancies between historical laboratory submissions and intentions to submit future cases for diagnostics call into question whether intention will translate into action on the part of FVSs. Additionally when there is a lack of common case selection principles driving sample submission, data generated by laboratories are unlikely to allow for reliable pattern recognition and it is difficult to determine the significance of changes to numbers of submissions or confirmed disease cases to the population [9], [11].

The lack of consensus among participants concerning the value of diagnostics, in particular characteristics of clinical cases that would dictate a need for laboratory support, highlights a knowledge deficit. Targeted efforts to improve the likelihood that FVSs would recognize animal health-related events that could represent an EID risk and perceive the need for diagnostics would be an important first step in improving the quantity, quality, and reliability of laboratory data that is an integral component of Sri Lanka’s ongoing disease surveillance efforts. It is worth emphasizing that though laboratory capacity in Sri Lanka is limited, and may not be able to identify the etiology of an EID event, previous experience with EIDs demonstrates that existing laboratory capacity is important to ruling out common causes of disease. For example, recognition of the 1995 Ebola virus outbreak in the Congo was delayed by a concurrent outbreak of *Shigella*, and it has been noted that the ability to rule out *Shigella* in cases of bloody diarrhea at the local level would have been equally as useful as the ability to rule in Ebola virus [45].

Participants used clinical history and examination findings rather than diagnostics to arrive at a diagnosis and guide treatment and were confident in their approach. Though some employed a hypothetico-deductive method to generate differential diagnoses, many used a pattern recognition method based on previous experience to recognize patterns in clinical characteristics that accompany a disease condition. They emphasized that the value of their diagnostic process was in its ability to inform treatment as opposed to aid in making a clinical diagnosis. In addition, response to therapy informed participants’ future diagnostic process and treatment decision-making. This approach has implications for EID recognition at the level of an individual FVS. A review of EID events in farm animals found that many events were detected when a clinician was unable to link clinical signs with a known disease, or when a clinician noted outbreaks of unusually severe clinical signs [24]. Therefore, if FVS focus is toward the treatment most appropriate given the clinical case presentation, and there is little consideration given to whether the clinical presentation represents something out of the ordinary at the level of the population, EID events could go undetected by individual FVSs until a time when there is widespread disease and less dramatic events could go overlooked altogether. As in the majority of cases participants decided not to submit samples, efforts to improve laboratory capacity and access are unlikely to impact this broader challenge to laboratory-based surveillance.

Based on the challenges to EID event detection by laboratories and individual FVSs in Sri Lanka, the argument can be made that surveillance methods that collect clinical case data from FVSs, and potentially other animal health care workers, could prove essential to EID event detection in Sri Lanka. Data collected could provide a population perspective on the burden of clinical syndromes and diagnoses in domestic animals that currently does not exist. It could allow decision makers to move away from relying solely on individual reports from FVSs and laboratories. The data could be combined with that from laboratories to inform a variety of animal health-related activities, from EID surveillance to FVS training to upgrades to infrastructure [35].

Equipping farmers with the ability to better recognize animal health-related events of potential significance and an understanding of the potential impact of missed events could be of benefit. Additionally, some of the barriers that deter farmers from utilizing government veterinary services in Sri Lanka could be addressed: though participants indicated that farmers contact them when they have a sick animal, the economic status of farmers in combination with the costs associated with veterinary and laboratory services as reported by participants may mean that animal health-related events are not always brought to the attention of a FVS. Incentives for farmers to engage the veterinary profession could be coupled with education to increase overall contact between FVSs and farmers and make communication during an animal health-related event more likely.

### Field Veterinary Surgeons and Surveillance

Surveillance is a public health practice undertaken by people in a wide range of contexts. Its practice is directly related to the environment in which it takes place and therefore a socio-ecological approach to analysis is warranted. Bronfenbrenner’s (1979) Ecological Systems Theory identifies five levels of influence on human behaviour (individual, interpersonal, organizational, community, and societal) that overlap and taken together comprise the environment in which human behaviours take place [46]. An assumption inherent to the socio-ecological approach is that interventions that operate at multiple levels are more effective in comparison to those that operate on a single level.

#### Individual-level influences on surveillance

The individual level in the socio-ecological model emphasizes the importance of characteristics of the individuals to intervention strategies. Participants identified surveillance as being important to situational awareness, but many overlooked its public health significance and the decisions and responsive actions it could serve to inform. Educating FVSs about the fundamentals of the surveillance process would be of benefit.

#### Interpersonal-level influences on surveillance

The interpersonal level in the socio-ecological model emphasizes the importance of social norms and social influences to intervention strategies. Participants have benefitted from the establishment of VICs in Sri Lanka but not because of the increased laboratory capacity *per se*: they contact the VIO when they feel it is warranted, either to refer or consult on a case. It is interesting to note that some participants have explored alternative, more proximate, sources of laboratory support. A starting point to improve efforts to engage FVSs in future surveillance initiatives would be to strengthen existing networks of communication. One could speculate that by promoting avenues of collaboration and communication, information concerning potential EID events could be transmitted among FVSs and other health care professionals more quickly and participation in surveillance initiatives would be supported within the veterinary profession [47]–[49]. This approach would be feasible in Sri Lanka as it relies on existing human resources within the community [47]–[49].

#### Organizational-level influences on surveillance

The organizational level in the socio-ecological model recognizes that changing the policies and practices of a workplace can serve to support behavioural change. In Sri Lanka, providing VIOs with the means to support FVS activities, as opposed to focusing solely on enhancing the laboratory capacity of VICs, is one form of incentive that remains unexplored. Stronger networks would support such efforts through dissemination of current data and information and reinforcement of learning objectives, as well as provide a continuous means of encouraging participation in surveillance [47].

#### Community-level influences on surveillance

The community level in the socio-ecological model recognizes that coordinating the efforts of members of a community is necessary to bring about change. The willingness of participants to engage in surveillance highlights that FVSs are underutilized in EID event detection. However, the lack of shared view on incentives for participation remains a challenge. Incentives will need to satisfy the motivations of a range of individuals. Future surveillance programs should consider some form financial compensation for the time dedicated by FVSs, along with infrastructure support and data feedback, so FVSs are able to see the benefits of their efforts [47]. Program administrators will need to demonstrate to FVSs that dedicating time and effort to surveillance is worthwhile and the outcomes are significant to farmers and the veterinary profession.

#### Societal-level influences on surveillance

The societal level in the socio-ecological model recognizes that there are societal or cultural high-level factors that create a climate that encourages or discourages behaviours. Broadly speaking, governments and the public health community create a climate that impacts ability and willingness of health care workers to report potential EID events. This process operates at the level of nations, animal health care workers, and farmers. In order to reap the benefits of efforts to network and educate farmers and FVSs in Sri Lanka, these individuals will need to feel empowered to report clinically suspect situations in animals to those with the ability to act, and rewarded for their efforts. Fears of negative consequences of reporting will need to be addressed. Previous research has shown that punishment for animal health-related event reporting needs to be avoided as it undermines efforts to engage veterinarians and farmers in surveillance [50]. One of the major challenges in Sri Lanka is the deficiency in transportation infrastructure: the state of the rail and road network and availability of transport deters farmers and FVSs from travelling. The results indicate that this infrastructure deficit is a significant barrier to surveillance and could undermine efforts at other socio-ecological levels.

Reporting of EID events is essential to protecting public health. In animal health, surveillance systems rely heavily on people to recognize and report incidents that could indicate an EID event. In this study, we describe utilisation of laboratories by FVSs in Sri Lanka and their perceptions of surveillance, with emphasis on their willingness to participate in various programs. From our findings we make recommendations to improve EID surveillance in Sri Lanka. Our experience demonstrates that an understanding of the human dimension of surveillance can enhance future efforts to detect EID events.
